# Case report: complete long-lasting response to multimodal third line treatment with neurosurgical resection, carmustine wafer implantation and dabrafenib plus trametinib in a *BRAFV600E* mutated high-grade glioma

**DOI:** 10.3389/fonc.2024.1359093

**Published:** 2024-05-07

**Authors:** Barbara Castelli, Marco Tellini, Melina Guidi, Marco Di Nicola, Laura Giunti, Anna Maria Buccoliero, Maria Luigia Censullo, Alessandro Iacono, Isacco Desideri, Lorenzo Genitori, Iacopo Sardi, Carla Fonte

**Affiliations:** ^1^ Neuro-oncology Department, Meyer Children’s Hospital IRCCS, Florence, Italy; ^2^ Pathology Department, Meyer Children’s Hospital IRCCS, Florence, Italy; ^3^ Radiology Department, Meyer Children’s Hospital IRCCS, Florence, Italy; ^4^ Radiotherapy Department, Careggi Hospital, Florence, Italy; ^5^ Neurosurgery Department, Meyer Children’s Hospital IRCCS, Florence, Italy

**Keywords:** high-grade glioma, MEK inhibitors, target therapy, dabrafenib, trametinib, pleomorphic xanthoastrocytoma, BRAFV600E, BRAF inhibitors

## Abstract

Dabrafenib plus trametinib is a promising new therapy for patients affected by *BRAFV600E*-mutant glioma, with high overall response and manageable toxicity. We described a complete and long-lasting response in a case of recurrent anaplastic pleomorphic xanthoastrocytoma CNS WHO-grade 3 *BRAFV600E* mutated. Due to very poor prognosis, there are a few described cases of high-grade glioma (HGG) patients treated with the combined target therapy as third-line treatment. The emergence of optimized sequencing strategies and targeted agents, including multimodal and systemic therapy with dabrafenib plus trametinib, will continue to broaden personalized therapy in HGG improving patient outcomes.

## Graphical Abstract

Timeline.

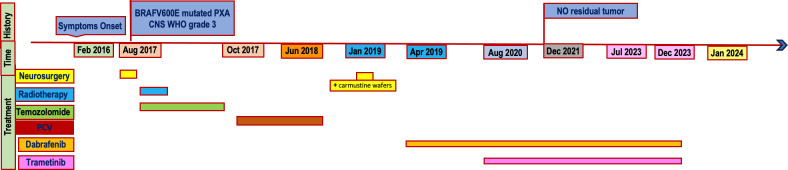

## 1 Introduction

High-grade gliomas (HGGs), tumors of neuroepithelial origin (1), represent the most common primary intracranial tumor in adults (2, 3). Differently, low- grade gliomas (LGGs) predominate in children (4, 5).

HGGs display a dismal prognosis despite surgical and chemo radiotherapeutic advances (1) and standard of care is commonly not curative. Throughout the understanding of molecular basis of tumors and recent insights, survival outcomes modestly increased, however, remaining limited and challenging. Therefore, worldwide researches are moving towards new frontiers and ongoing trials are investigating novel targeted agents (1). In the last years, important advances in the field of molecular biology and pathology have been accomplished (6).

MAPK (mitogen-activated protein kinase) pathway, implicated in carcinogenesis, has been found altered in most glial tumors (7, 8), promoting cellular overgrowth and overcoming metabolic stress (9). The pathway includes a small G protein (RAS) and three protein kinases in a downstream signaling pathway (respectively RAF – composed of A-RAF, B-RAF and RAF-1 or C-RAF kinases, MEK – composed of MEK1 and MEK2, ERK – composed of ERK1 and ERK2) (10, 11). ERK (extracellular signal-regulated kinase) is a MAPK that functions as the major effector of the RAS oncoprotein, translocating to the nucleus to activate transcription factors (10). Driving oncogenic mutations should develop upstream of the MAPK pathway (11).

Most *BRAF* variants are missense mutations at amino acid position 600, resulting in an exchange of valine for glutamate (referred to as *BRAFV600E*) (12). Activating *BRAFV600E* kinase mutations occur in ~7% of human malignancies (13). Initially described in melanoma, colon and papillary thyroid carcinoma, these alterations have also been observed in primary nervous system tumors (14). High mutation frequencies have been detected in pleomorphic xanthoastrocytomas (PXA), gangliogliomas and extra-cerebellar pilocytic astrocytomas (14), but the mutation has also been found in others HGGs (12), in particular in epithelioid glioblastoma (15).

The BRAF inhibitors vemurafenib, dabrafenib and encorafenib selectively target BRAF kinase, interfering with MAPK signaling pathway (16). Selumetinib and trametinib are MEK inhibitors (MEKi) (7). The combination of BRAF and MEK inhibitor have been approved in various cancers by the US Food and Drugs Administration (FDA) (17) and the European Medicines Agency (EMA). It is known that the blockage of two downstream pathway components with dual BRAF/MEK inhibition may improve tumor control and patient survival (18).

Recently, MEK inhibitors and BRAF inhibitors have been successfully used in pediatric LGG patients (19), with a relatively well-tolerated side effect profile (1). Few data are available on their efficacy in relapsing refractory HGGs.

Herein we report a case of complete long-lasting response to combined dabrafenib/trametinib as third-line therapy in a patient with frontal HGG.

## 2 Case report

**Figure 1:**
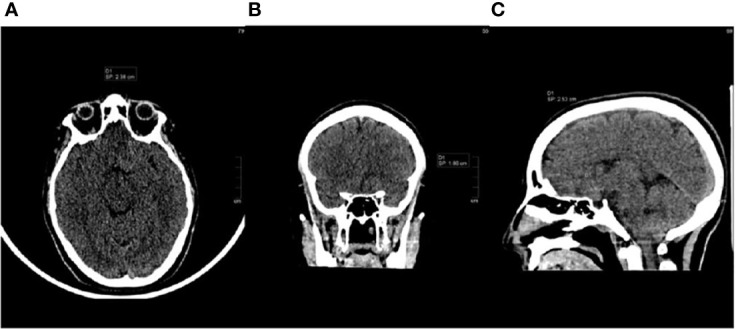
Brain CT scan at diagnosis, August 2016 [**(A)**: axial, **(B)**: coronal, **(C)**: sagittal].

**Figure 2:**
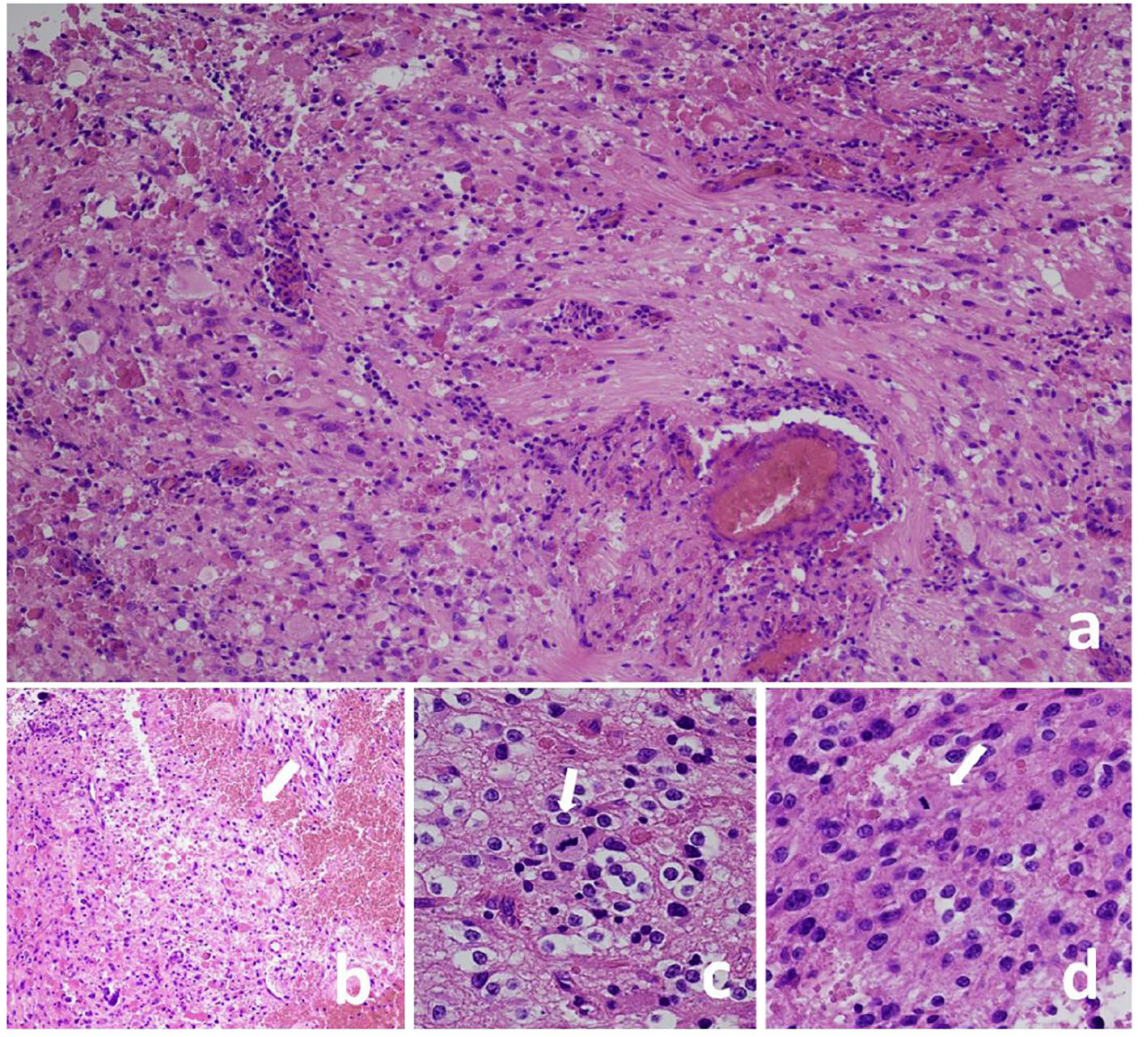
Pleomorphic xanthoastrocytoma, CNS WHO grade 3, lesion composed of pleomorphic cells **(A)** and oligodendrocyte-like cells **(C)**. Perivascular lymphocyte cuffing and granular bodies are present **(A)** as well as necrosis [**(B)**, arrow] and mitoses [**(C, D)**, arrows]. Hematoxylin and eosin stain **(A-D)**; Original magnification: a-b 10 X, c 40 X, d 20 X.

In February 2016, a 21-year-old white female presented her first seizure episode. In August 2016 she was admitted to Anna Meyer Children’s Hospital IRCCS in Florence for recurrent episodes. Imaging revealed a left frontal lesion (**Figure 1**). A partial resection was performed. The histological examination diagnosed anaplastic PXA *BRAFV600E* mutated CNS WHO-grade 3. The lesion was composed of pleomorphic, xanthomatous and oligodendrocyte-like cells. Perivascular lymphocytic cuffing and numerous granular bodies were present. Mitoses (more than 5 X 10 HPF) and necrosis were seen (**Figure 2**). At immunohistochemistry GFAP, CD34 and *BRAF* p.V600E resulted positive; rare cells expressed synaptophysin. Molecular study confirmed *BRAF* p.V600E mutation (c. 1799T>A) whereas FISH analysis documented homozygous deletion of *CDKN2A*.

**Figure 3:**
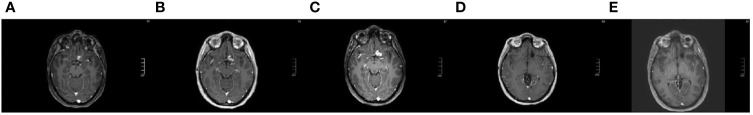
Axial T1 contrast-enhanced brain MRI [**(A)**: after first line therapy, January 2017; **(B)**: at first progression, October 2017; **(C)**: at second progression, presurgical, December 2018; **(D)**: complete response during target therapy, December 2021; **(E)**: persistence complete response one month after target therapy interruption, January 2024].

From October 2016 to December 2016 a volumetric modulated radiotherapy course was delivered for a total dose of 59,4 Gy in 33 fractions with concomitant and adjuvant temozolomide therapy (Stupp regimen). However, O6-methylguanine-DNA methyltransferase (MGMT) promoter was not methylated. Brain MRI at the end of radiotherapy revealed residual disease (**Figure 3A**).

In October 2017 (14 months after first surgical resection), a brain MRI showed progressive disease next to the resected area (first progression, **Figure 3B**), therefore six courses of chemotherapy with procarbazine, lomustine and vincristine (PCV) were administered (the last in June 2018) with disease control.

In January 2019 (7 months after the end of second line treatment) a cranial MRI showed progression of disease (second progression, **Figure 3C**) and another neurosurgical partial resection with carmustine wafers implantation was performed. The histological analysis confirmed the previous diagnosis. Considering the residual disease, in April 2019 the 24-year-old female patient with *BRAF* mutated anaplastic PXA started third-line therapy with dabrafenib. In August 2019 she suffered from Herpes Zoster reactivation, leading to temporary target drug suspension. The well-known tumor residue was less evident on the subsequent MRIs performed every three/four months. Given the literature data of the most effectiveness with better tolerability and the reduced possibility of resistance (13, 20–22), in August 2020 the patient started combination treatment with dabrafenib plus trametinib. Temporary interruption was required for pyrexia and in September 2020 for the occurrence of erythema nodosum grade 3 Common Terminology Criteria for Adverse Events (CTCAE) v.4. Dabrafenib and trametinib were then continued at a reduced dose (25%-50% reduction). The combined therapy was overall well tolerated. Since December 2021 the residual tumor has not been longer visible (**Figure 3D**). MRI evaluation, performed on July 27^th,^2023, showed no recurrence of the disease, three years after BRAF/MEK inhibitor combination treatment beginning. In December 2023, considering the optimal response and the reported toxicity, the dual target treatment was interrupted. Last MRI, performed on January 29^th^, 2024 (one month after drug cessation, 5 years after second progression) revealed persistent complete response (**Figure 3E**).

## 3 Discussion and conclusion

PXA is a tumor with a wide range of morphology (19). Two WHO grades (CNS WHO 2 or 3) are assigned, based on a mitotic count of more than 5 mitoses per 10 microscopic high power fields (19). Grade 3 includes the anaplastic variant (23). Anaplastic PXA is associated with poorer clinical outcomes compared with PXA CNS WHO 2 (24). Anaplastic variant of PXA shows histological characteristics as well as clinical course comparable with Grade 3 astrocytoma (25). Gross total resection should be the goal of initial treatment and it remains unclear whether adjuvant radiation and chemotherapy are able to prevent progression or dissemination (24). Early disease recurrence in anaplastic PXA is associated with fatal outcomes (25). *BRAFV600E* mutation can be detected in up to 70% of these tumors, combined with *CDKN2A* homozygous deletion in greater than 90% (19). Considering the emerging molecular landscape and the frequent failure of conventional therapies, novel therapeutic strategies are under investigation in the treatment of HGGs.

Targeted therapies, including mutant BRAF inhibitors (dabrafenib) and MEK inhibitors (trametinib), have yet shown promising results in other cancers refractory to conventional chemotherapy (26). The safety and effectiveness of MEKi treatment have also been established in improving symptomatology and quality of life in patients affected by plexiform neurofibromas in Neurofibromatosis Type I (7). Considering brain tumors, MAPK inhibitors have shown encouraging results in LGG showing alterations of this pathway. Dabrafenib demonstrated meaningful clinical activity and acceptable tolerability in patients with *BRAFV600*-mutant LGG (27). Trametinib was an active and feasible treatment for progressive pediatric MAPK-aberrant LGGs, leading to disease control (28). Recently, the Food and Drug Administration (FDA) approved dabrafenib in combination with trametinib for the treatment of pediatric *BRAFV600E* LGG (29). Instead, data are still limited on their efficacy in *BRAFV600E* mutated HGGs. In 2014 Robinson et al. described the first known case of complete response in a *BRAFV600E*-mutated HGG to vemurafenib (BRAF inhibitor) therapy (20). In 2022 Arbour et al. reported an 18-year-old female with a grade 3 PXA treated upfront with dabrafenib and trametinib and conducted a systematic literature review of patients with HGG and *BRAFV600E* mutations treated with BRAF inhibitors (30).

In a phase 2 Rare Oncology Agnostic Research (ROAR) basket trial (NCT02034110) Dabrafenib plus trametinib showed clinically meaningful activity in patients with BRAFV600E mutation-positive recurrent or refractory HGG: 15 (33%; 95% CI 20-49) of 45 patients had an objective response by investigator assessment, including three complete responses and 12 partial responses (31). Further ongoing studies are evaluating MEK inhibition also in HGG patients. An Open Label, multi-center Roll-over Study is assessing Long-term effect of BRAFV600E and MEK inhibition with dabrafenib and trametinib in a subset of HGG (NCT03975829) (1). A phase I/II Trial is designed to study the combination of Dabrafenib, Trametinib and Hydroxychloroquine for Patients with Recurrent LGG or HGG with a BRAF aberration (NCT04201457). Another phase II trial studies how well the combination of dabrafenib and trametinib after radiation therapy in children and young adults with *BRAF V600* mutated HGG (NCT03919071).

Our case report suggests that BRAF/MEK inhibition is a potential promising strategy also in the treatment of recurrent and refractory HGG, non-stable responsive to surgery, radiotherapy, first and second line chemotherapy. The patient on the third-line combined target therapy achieved even a complete extraordinary response, with disappearance of residual disease.

The patient started a therapy with BRAF and MEK inhibitors on the basis that previous studies on melanoma suggested the possibility of resistance (13, 20), Moreover, Hargrave et al. in a phase II trial in pediatric relapsed/refractory *BRAFV600*–mutant HGG assessed tolerable safety and durable responses of the combined therapy, compared to traditional chemotherapy (32). Hypotheses for mechanisms of acquired resistance to BRAF inhibition include secondary mutations in *BRAF*, MAPK reactivation, and activation of alternative survival pathways (13). Reports in colorectal cancer suggest *BRAF*-mutant tumors may escape inhibition by amplifying receptor tyrosine kinases (20, 33), Additionally, combination of MEK and BRAF inhibitors reduces squamous cell carcinoma risk observed with BRAF inhibitors monotherapy (1). Combined treatment is reported to be well tolerated with mostly moderate and reversible side effects (21). In an open-label study involving patients with metastatic melanoma with *BRAFV600* mutations, dabrafenib and trametinib were safety combined at full monotherapy doses, with significatively improvement of progression-free survival (22). In our case in combined therapy temporary interruption was required in two events: pyrexia and for the occurrence of erythema nodosum, recurred some months later. Dabrafenib was then continued at a reduced dose (25% reduction) and the combined therapy was overall well tolerated.

Data on long-term response are still poor. Our case report describes an extremely great 3-year persistent response on combined target therapy. We must take into account that the combined target therapy was a component of a multimodal approach including neurosurgery and carmustine wafers implantation (CW). Approved to treat newly or recurrent HGG, CW efficacy was reported doubtful: CW may provide a therapeutic coverage during the usual radiotherapy delay of 2 to 6 weeks (34). In our case, the optimal neuro radiological response was observed at almost two years since CW implantation, therefore it was most likely related to the dual target treatment. However, CW was a part of the third line therapy, thus composed of a multimodal approach.

Despite promising preclinical and clinical trials, several issues persist (1). Disease control after MEKi withdrawal was not sustained in a fraction of patients (28). Even on temporary effect, therapeutic goals could include extending survival and improving quality of life in patients with relapsed disease (20). CNS tumors with alternative *BRAF* alterations, such as alternate *V600* mutations or *BRAF* fusions, may differently respond to target therapy (20): for example it is important to note that BRAF inhibitor therapy in patients with *BRAF* gene fusion or duplications activates the MAPK signaling pathway in cells with wild-type *BRAF* at *V600* (27), therefore in this setting MEK inhibitors represent the strategy of choice (35).

Moreover, several studies are investigating the use of targeted therapy as a first-line treatment (26), which could open extraordinary perspectives.

Long term follow up would supply data on disease evolution after treatment discontinuation and further studies are expected to provide standardized treatment duration indications.

In conclusion, our case report suggests that BRAF/MEK inhibition may represent a potential therapeutic strategy also in patients with refractory relapsing HGGs *BRAF* mutated, not responsive to conventional therapies. The achieved complete response in a recurrent disease is an exceptional reached goal. The long-lasting response is also of great importance, giving long-term insights in combined target therapy. However, this is a limited study, reporting our favorable experience only in a single patient. Further studies are ongoing and more data on larger cohorts are needed to clarify present issues. Despite this exciting result, ongoing prospective studies will determine whether dabrafenib and trametinib combination can improve relapsed HGGs *BRAF* mutated outcomes.

## Data availability statement

The original contributions presented in the study are included in the article/supplementary material. Further inquiries can be directed to the corresponding author.

## Ethics statement

The studies involving humans were approved by Comitato Etico Regione Toscana, Azienda Ospedaliera Universitaria Meyer IRCCS. The studies were conducted in accordance with the local legislation and institutional requirements. Written informed consent for participation was not required from the participants or the participants’ legal guardians/next of kin in accordance with the national legislation and institutional requirements. Written informed consent was obtained from the individual(s) for the publication of any potentially identifiable images or data included in this article.

## Author contributions

BC: Conceptualization, Data curation, Formal analysis, Methodology, Resources, Validation, Writing – original draft. MT: Writing – original draft. MG: Validation, Visualization, Writing – review & editing. MD: Validation, Visualization, Writing – review & editing. LaG: Resources, Validation, Writing – review & editing. AB: Resources, Validation, Writing – review & editing. MC: Visualization, Writing – review & editing. AI: Resources, Validation, Writing – review & editing. ID: Resources, Validation, Writing – review & editing. LG: Supervision, Validation, Visualization, Writing – review & editing. IS: Conceptualization, Data curation, Supervision, Validation, Visualization, Writing – review & editing. CF: Conceptualization, Data curation, Formal analysis, Methodology, Supervision, Writing – review & editing.
